# Does Religion Buffer Against the Detrimental Effect of Cyberbullying Victimization on Adults’ Health and Well-Being? Evidence from the 2014 Canadian General Social Survey

**DOI:** 10.1177/08862605211050092

**Published:** 2021-11-22

**Authors:** Lei Chai

**Affiliations:** 1 Department of Sociology, University of Toronto, Toronto, ON, Canada

**Keywords:** cyberbullying victimization, self-rated health, self-rated mental health, self-rated life satisfaction, religious service attendance, religious beliefs, Canada

## Abstract

While prior research has well-documented the detrimental effect of cyberbullying victimization on health and well-being among children and adolescents, less is known about whether the same adverse pattern can be observed among adults. Moreover, it is unclear about what psychosocial resources might moderate this association. The present study uses a nationally representative cross-sectional survey—2014 Canadian General Social Survey (*N* = 17,548)—to examine three research questions. First, is cyberbullying victimization associated with adults’ self-rated health, mental health, and life satisfaction? Second, how does religiosity—religious service attendance and religious beliefs—moderate this association? Third, do any observed patterns further differ for men and women? Through a series of logistic and ordinary least squares regression models, the results show that adults who experienced cyberbullying victimization in the past 5 years are more likely to report poor self-rated health and mental health compared to those who did not experience cyberbullying victimization in the past 5 years. Likewise, cyberbullying victimization is also associated with lower levels of life satisfaction. In addition, the adverse associations of cyberbullying victimization in the past 5 years with self-rated health and life satisfaction are weaker among those who attended religious service at least once a week in the past twelve months. A similar pattern is observed for the buffering effect of viewing religious beliefs as very important in the adverse association of cyberbullying victimization in the past 5 years with self-rated life satisfaction. There is also evidence suggesting the gendered buffering effect of the importance of religious beliefs in the association between cyberbullying victimization and self-rated health. This study makes important empirical and theoretical contributions to the growing field of research on the association between cyberbullying victimization and health and well-being and to our understanding of how religion matters to individuals dealing with stressful experiences.

## Introduction

With ongoing technological developments and increasing accessibility to the Internet, cyberbullying has emerged as a significant public health concern affecting children and adolescents (Chester et al., 2019). Cyberbullying refers to “any behavior performed through electronic or digital media by individuals or groups that repeatedly communicates hostile or aggressive messages intended to inflict harm or discomfort on others” (Tokunaga, 2010, p. 278). Due in part to the anonymity of the perpetrator, the victim might feel helpless not being able to identify the perpetrator, and the sense of anonymity on the part of the perpetrator might also contribute to increased hostility and reduced empathy towards the victim (Aboujaoude et al., 2015; Müller et al., 2018). Coupled with other unique challenges faced by the victim including an infinite audience and a lack of physical constraints from the perpetrator (Patchin & Hinduja, 2011; Payne & Hutzell, 2017), cyberbullying might be more harmful to health and well-being compared to traditional bullying. Past research shows that victims of cyberbullying are associated with worse internalizing and externalizing symptoms compared to victims of traditional bullying (Schneider et al., 2012; Waasdorp & Bradshaw, 2015). Nevertheless, it is important to acknowledge that there is considerable overlap between traditional bullying and cyberbullying (Wang, Musumari, et al., 2019), research shows that victims of both traditional and cyberbullying are associated with greater health disparities compared to those who are victims of either form of bullying (Schneider et al., 2012).

While prior research has well established the detrimental health and well-being consequences of cyberbullying victimization among children and adolescents (Bonanno & Hymel, 2013; Chai et al., 2020; Cole et al., 2016; Fisher et al., 2016; Hinduja & Patchin, 2019; Kowalski & Limber, 2013; Kowalski et al., 2014; Martínez-Monteagudo et al., 2020; Perret et al., 2020; Ranney et al., 2016; Torres et al., 2020; Vaillancourt et al., 2017; Vieno et al., 2015), it remains unclear about whether the same adverse pattern can be observed among adults. Despite the potential adverse consequences of cyberbullying victimization (Chu et al., 2018; Wang, 2021), it is plausible that many adults might have heard or experienced cyberbullying when they were younger, which would give them an advantage over children and adolescents. Coupled with more developed cognitive and coping skills, adults might consider cyberbullying victimization as a temporary and normal part of life and therefore feel less threatened. Surprisingly, no research to my best knowledge has used nationally representative samples to assess the association between cyberbullying victimization and multiple health and well-being outcomes among adults simultaneously. Furthermore, social resources often buffer the detrimental consequences of stress (Pearlin & Bierman, 2013). An extensive research has established that religion is beneficial for people’s health and well-being (Das & Nairn, 2016; Elliott & Hayward, 2009). In this study, I shift the focus to examine the moderating potential of religion in the relationship between cyberbullying victimization and health and well-being. Although there is evidence that religion attenuates the detrimental effect of social stressors on health and well-being (e.g. Bierman et al., 2018; Jung, 2014), no studies that I am aware of have explored its moderating potential within the context of cyberbullying.

With these gaps in mind, the present study selects Canadian adults (*N* = 17,548) from a nationally representative sample—the 2014 Canadian General Social Survey—to examine three research questions. First, is cyberbullying victimization associated with adults’ self-rated health, mental health, and life satisfaction? Second, how does religiosity—religious service attendance and religious beliefs—moderate this association? Third, do any observed patterns further differ for men and women?

## Background

### Cyberbullying Victimization and Health and Well-being

Aligned with the prediction of the stress process model (Pearlin & Bierman, 2013), cyberbullying can be conceptualized as a prominent social stressor (Chu et al., 2018; Wang, 2021), which limits one’s capacity to access coping resources, thereby contributing to adverse health and well-being consequences. Past research has found evidence supporting this claim among children and adolescents. For instance, studies have shown that cyberbullying victimization is associated with higher levels of mental health problems, such as anxiety (Jenaro et al., 2021), depression (Chu et al., 2018; Jenaro et al., 2021; Wang, Xie, et al., 2019; Zhu et al., 2019), posttraumatic stress disorder (PTSD) (Ranney et al., 2016), and suicidal ideation (Mitchell et al., 2018; Tokunaga, 2010). Similar patterns have also been observed for physical health problems, such as self-rated health (Chai et al., 2020; Zhu et al., 2019), sleep problems (Kowalski & Limber, 2013), headaches (Kowalski & Limber, 2013), and stomach pain (Kowalski & Limber, 2013). There is also evidence that cyberbullying victimization is associated with lower levels of life satisfaction (Chai et al., 2020; Salazar, 2017).

Despite these valuable empirical findings, surprisingly little is known about the extent to which cyberbullying victimization shapes adults’ health and well-being. Most studies that document the consequences of cyberbullying victimization among adults have focused on work outcomes. For instance, employees who experienced cyberbullying victimization are more likely to report absenteeism (Kowalski et al., 2018), intention to resign (Baruch, 2005), and lower levels of job satisfaction (Baruch, 2005; Coyne et al., 2017; Farley et al., 2015; Snyman & Loh, 2015), and job performance (Baruch, 2005). Among the only two studies, to my best knowledge, that examine the association between cyberbullying victimization and adults’ health, the first shows that cyberbullying victimization among medical doctors is linked to elevated levels of mental strain (Farley et al., 2015). The second study, using a nationally representative survey, reveals that cyberbullying victimization is linked to worse mental health, everyday limitations due to mental health problems, and elevated binge drinking and drug use (Kim et al., 2017). Based on these theoretical ideas embedded in the stress process model and empirical evidence, it is reasonable to speculate that adults who experienced cyberbullying victimization will be associated with poorer self-rated health, mental health, and life satisfaction than their counterparts who did not experience cyberbullying victimization (**Hypothesis 1**).

### The Buffering Effect of Religion

Religion has been portrayed as an effective psychosocial resource that weakens the adverse consequences of stressful events and chronic stressors (Bierman, 2006; Bierman et al., 2018; Bradshaw & Ellison, 2010; Ellison et al., 2019; Hastings & Roeser, 2020; Jung, 2014; Shah, 2019). Despite its complexity and multidimensionality, religion can be divided into two broad categories, including organizational involvement (e.g. religious service attendance) and nonorganizational involvement (e.g. religious beliefs) (Bierman et al., 2018; Bradshaw & Ellison, 2010). Higher organizational and nonorganizational involvement might buffer the negative consequences of cyberbullying victimization on health and well-being (Bradshaw & Ellison, 2010).

On the one hand, research suggests that religious service attendance can reduce the harmful effect of social stressors in several ways (Bradshaw & Ellison, 2010; Hill et al., 2006; Jung, 2021). First, religious service attendance provides an opportunity to develop social networks where individuals receive social support and friendship from the fellow church members (Bradshaw & Ellison, 2010; Jung, 2021). A feeling of not being alone might lead to cyberbullying seeming less threatening and stressful. Second, self-care has been a primary goal of many religious communities, and greater religious service attendance can promote a number of health behaviors (Hill et al., 2006). Behaviors like regular physical exercise and moderate drinking or/and smoking might therefore benefit victims’ health and well-being. Third, religious service attendance might offer activities that help members bolster feelings of mastery and self-esteem that are eroded by experiences of cyberbullying victimization. Based on these theoretical ideas, religious service attendance might therefore attenuate the detrimental effect of cyberbullying victimization on health and well-being.

On the other hand, religious beliefs could also provide a similar function (Bradshaw & Ellison, 2010; Gorsuch & Hao, 1993; Jung, 2014; Krause, 2005). First, religious beliefs can shape people’s worldviews, that is, people might reinterpret stressful events or chronic stressors as a grand divine plan (e.g. God) (Jung, 2014). Viewing cyberbullying victimization as a part of God’s plan might reduce its stressfulness and ultimately improve health and well-being. Second, individuals with greater religious beliefs are more likely to develop a personal connection with God (Bradshaw & Ellison, 2010). This kind of perception might in turn provide victims of cyberbullying a sense of comfort and reassurance by shifting their attention away from personal struggles and focusing on spiritual matters. Third, it is evident that religious beliefs and willingness to forgive are highly correlated (Gorsuch & Hao, 1993). Individuals with greater religious beliefs might let the anger and resentment go that arises from experiences of cyberbullying victimization and lead to adverse health and well-being consequences. Despite a lack of empirical evidence addressing the potential moderating effect of religion in relation to cyberbullying victimization, past research has established that religious service attendance and religious beliefs buffer against the association of stressors (e.g. discrimination, unemployment, or financial strain) with health consequences (Bradshaw & Ellison, 2010; Hastings & Roeser, 2020; Shah, 2019). Together, these studies might suggest that the detrimental effect of cyberbullying victimization on self-rated health, mental health, and life satisfaction will be weaker for adults who frequently attended religious services and who viewed religious beliefs as more important, respectively (**Hypothesis 2**).

### Gender Differences

There are reasons to speculate that the moderating effect of religion might further differ for men and women. The gender role socialization perspective is a useful framework that provides a rationale for the proposed relationship (Bierman et al., 2018; Jung, 2014, 2020). According to the gender role socialization perspective, “[t]he socialization of females tends to stress submission, nurturance, and caretaking whereas men are socialized to value aggressiveness and independence” (Jung, 2014, p. 1129). Due to these gendered norms and expectations, women are more likely than men to develop extensive social ties and interpersonal skills within religious settings (Krause et al., 2002; McFarland, 2010), thereby receiving greater social support (Krause et al., 2002). Thus, women who frequently attend religious services might benefit more from social resources offered by religious communities than men.

By contrast, masculinity embedded in traditional gender norms prevent men from receiving social support from fellow members because they are expected not to depend on external resources to cope with personal life struggles (Bennett, 2007). As scholars have stressed, “[m]en are therefore likely to be less reliant on not only the social network provided by religious attendance but also less dependent on support from a higher power or religious forces that supersede independence or personal control and also more resistant to religious forms of social control” (Bierman et al., 2018, p. 938).

Taken together, women might experience greater coping efficacy from both religious service attendance and religious beliefs. Past research, however, has primarily focused on the moderating potential of religious service attendance. For instance, Bierman and colleagues (2018) show that regular religious service attendance only moderates the association between discrimination and sleep problems for women, but not for men. Likewise, Jung (2014) indicates that the adverse effect of stress on happiness is weaker only among women with more frequent religious service attendance. A more recent study also demonstrates a similar pattern, suggesting that religious service attendance shows a buffering role in the association between perceived distributive unfairness and depression for women, but not for men (Jung, 2021). Despite a lack of empirical evidence on the moderating effect of religious beliefs, these theoretical ideas embedded in the gendered socialization perspective suggest that the buffering effects of religious service attendance and the importance of religious beliefs are stronger for women than men, respectively (**Hypothesis 3**).

## Methods

### Data and Sample

The present study used data from Statistics Canada’s, 2014 General Social Survey (GSS-28). The GSS is a cross-sectional survey conducted annually where it collects data on social trends and monitors the living conditions and well-being of Canadians over time. Each cycle of the GSS has a thematic focus; Cycle 28 focuses on victimization. The target population of the GSS-28 included Canadians aged 15 years and older. Data were collected primarily from respondents through telephone interviews. However, the GSS-28 also offered respondents an internet-based option and a face-to-face option for those living in the territories. See Statistics Canada (2014) for further details about GSS sample design and data collection procedures. The response rate was 52.9%. Given the primary interest of this study was on adults, I excluded the youngest age category of 15–24 from all the analyses. I also removed individuals who had never used the internet over the past 5 years. The final sample for this study was 17,548 (7,769 men and 9,779 women).

### Measures

I analyzed three outcome variables: self-rated health, self-rated mental health, and self-rated life satisfaction. *Self-rated health* was measured based on the question: “In general, would you say your health is…?” The responses included “excellent,” “very good,” “good,” “fair,” and “poor.” Past research shows that self-rated health is highly correlated with more objective measures of health (e.g., morbidity and mortality) (Ferraro et al., 1997; Idler & Benyamini, 1997). There is also considerable evidence that self-rated health predicts outcomes more accurately than physician diagnoses (DeSalvo et al., 2006; Jylhä, 2009). I recoded the item into a dummy variable where “fair/poor” was coded as “1” and “excellent/very good/good” was coded as “0” (reference) (Chai et al., 2020; Huang et al., 2021).

*Self-rated mental health* was measured based on the question: “In general, would you say your mental health is…?” The responses included “excellent,” “very good,” “good,” “fair,” and “poor.” Past research suggests that self-rated mental health is a useful measure of overall mental health. Self-reported mental health and a variety of mental disorders (e.g., depression, psychological distress) are highly correlated, though it is not a substitute measure of specific disorders (Chai & Xue, 2021; Wu & Schimmele, 2021). Moreover, this single-item measure of self-reported mental health can capture both mental illness and mental well-being (Ahmad et al., 2014; Wu & Schimmele, 2021). I recoded the item into a dummy variable where “fair/poor” was coded as “1” and “excellent/very good/good” was coded as “0” (reference) (Chai et al., 2020; Sivakumaran & Margolis, 2020).

*Self-rated life satisfaction* was measured based on the question: “how do you feel about your life as a whole right now?” Responses were recoded from “0” (very satisfied) to “10” (very dissatisfied). Similar to self-rated health and mental health, prior research has shown that this single item is a reliable and valid measure of general well-being (Chai et al., 2020; Jovanović & Lazić, 2020).

My primary predictor variable was experiences of *cyberbullying victimization*. Cyberbullying victimization was assessed based on the following questions: In the past 5 years, “have you ever received threatening or aggressive e-emails or instant messages where you were the only recipient?” “have you ever been the target of threatening or aggressive comments spread through group e-mails, instant messages or postings on internet sites?” “has anyone ever sent out or posted pictures that embarrassed you or made you feel threatened?” “has anyone ever used your identity to send out or post embarrassing or threatening information?” and, “have you ever been the target of any other kind of cyber stalking/bullying (which is the use of the internet to embarrass, intimidate or threaten someone), not already mentioned?” Responses to each question included “yes” and “no.” The cyberbullying victimization category was coded as “1” when at least one item was reported as “yes,” while the category was coded as “0” (reference) when all bullying items were reported as “no.”

*Religiosity*, the central moderating variable, was assessed based on two items. *Religious service attendance* was measured based on the question: “Not counting events such as weddings or funerals, during the past 12 months, how often did you participate in religious activities or attend religious services or meetings?” Responses were recoded as “not at all” (reference), “once or twice a year,” “at least 3 times a year,” “at least once a month,” and “at least once a week.” *Importance of religious or spiritual beliefs* was measured based on the question: “How important are your religious or spiritual beliefs to the way you live your life? Would you say they are…?” Responses were recoded as “not at all important” (reference), “not very important,” “somewhat important,” and “very important.” The correlation between the two indicators (i.e., religious service attendance and the importance of religious beliefs) was 0.54, suggesting that they were only moderately correlated and needed to be examined separately.

*Gender* was coded as “male” (reference) and “female.”

*Control variables.* I controlled for the following socio-demographic characteristics: *Age group* was coded as “25-34” (reference), “35-44,” “45-54,” “55-64”, “65-74,” and “75 and above.” *Visible minority status* was coded as “visible minority” and “not a visible minority” (reference). *Marital status* was coded as “married” (reference), “living common-law,” “widowed,” “separated,” “divorced,” and “single, never married.” *Presence of children* was recoded as “yes” and “no” (reference). *Education* was recoded into five categories, including “less than high school,” “high school,” “some college/college” (reference), “BA,” and “post-graduate.” *Employment status* was assessed based on the question: “Did you have a job or were you self-employed at any time last week?” Responses included “yes” (reference) and “no.” *Household income* was coded as “less than $20,000,” “$20,000 to $39,999,” “$40,000 to $59,999,” “$60,000 to $79,999,” “$80,000 to $99,999,” “$100,000 to $119,999,” “$120,000 to $139,999,” and “$140,000 or more (reference).” *Region* included the following four categories: “Atlantic,” “Central” (reference), “Prairies,” and “West.” Table 1 presents the descriptive statistics of selected variables in the analyses.

**Table 1.** Descriptive statistics of selected variables in the analyses (means, percentages, and standard errors).

	Full sample		Men		Women	
	M/%	SE	M/%	SE	M/%	SE
Self-rated health						
Poor/fair	8.53		7.89		9.14	
Excellent/very good/good (reference)	91.47		92.11		90.86	
Self-rated mental health						
Poor/fair	3.84		3.33		4.26	
Excellent/very good/good (reference)	96.19		96.67		95.74	
Self-rated life satisfaction	1.53	.01	1.52	.02	1.53	.02
Cyberbullying victimization						
Yes	4.67		4.66		4.67	
No (reference)	95.33		95.34		95.33	
Religious service attendance						
Not at all (reference)	48.42		50.43		46.54	
Once or twice a year	16.86		17.61		16.16	
At least 3 time a year	10.96		10.29		11.58	
At least once a month	8.79		8.17		9.37	
At least once a week	14.97		13.50		16.35	
Importance of religious beliefs						
Not at all (reference)	22.33		27.84		17.18	
Not very important	15.53		16.48		14.65	
Somewhat important	31.29		30.23		32.28	
Very important	30.85		25.45		35.90	
Age group						
25–34 (reference)	21.54		21.41		21.66	
35–44	21.42		21.91		20.95	
45–54	20.53		20.54		20.53	
55–64	19.79		19.08		20.46	
65–74	11.84		11.87		11.81	
75 and above	4.87		5.19		4.58	
Visible minority						
Yes	12.10		12.03		12.17	
No (reference)	87.90		87.97		87.83	
Marital status						
Married (reference)	62.29		64.59		60.14	
Living common-law	13.89		14.04		13.75	
Widowed	3.21		1.46		4.86	
Separated	2.11		1.98		2.22	
Divorced	4.64		3.17		6.02	
Single, never married	13.86		14.76		13.02	
Presence of children						
Yes	42.03		41.19		42.83	
No (reference)	57.97		58.81		57.17	
Education						
Less than high school	6.81		7.53		6.13	
High school	22.94		22.99		22.89	
Some college/college (reference)	37.48		37.60		37.38	
BA	22.12		20.85		23.31	
Post-graduate	10.65		11.03		10.30	
Employment status						
Yes (reference)	69.52		74.76		64.61	
No	30.48		25.24		35.39	
Household income						
Less than $20,000	3.31		2.75		3.83	
$20,000 to $39,999	9.21		7.82		10.50	
$40,000 to $59,999	13.55		13.00		14.07	
$60,000 to $79,999	14.52		14.09		14.92	
$80,000 to $99,999	14.08		14.27		13.89	
$100,000 to $119,999	11.40		12.07		10.77	
$120,000 to $139,999	9.30		10.01		8.65	
$140,000 or more (reference)	24.63		25.99		23.36	
Region						
Atlantic	7.08		6.77		7.36	
Central (reference)	63.49		63.96		63.05	
Prairies	17.35		17.33		17.36	
West	12.09		11.93		12.23	
N	17,548		7,769		9,779	

*Note.* Ms/%s are weighted. The Ns are unweighted.

### Analysis Plan

I used logistic and OLS (ordinary least squares) regression models to estimate the associations among the focal variables, all models included the full set of control variables. In Table 2, Models 1–3 tested the direct association of cyberbullying victimization with self-rated health, mental health, and life satisfaction, respectively. Next, in Table 3, Models 1–6 examined whether the association of cyberbullying victimization with self-rated health, mental health, and life satisfaction differed across religious service attendance and the importance of religious beliefs. Finally, in Table 4, Models 1–6 tested gender differences in how religious service attendance and the importance of religious beliefs shaped the association of cyberbullying victimization with self-rated health, mental health, and life satisfaction. To account for the potential non-response bias, I added “weights” provided by Statistics Canada to all descriptive and multivariate analyses.

**Table 2.** Logistic and OLS regression models predicting self-rated health, mental health, and life satisfaction.

	Self-rated health		Self-rated mental health		Self-rated life satisfaction	
	Model 1		Model 2		Model 3	
	OR	SE	OR	SE	B	SE
Cyberbullying victimization (=1)	1.872***	.294	1.761**	.348	.395***	.074
Intercept	.173		.043		1.729	
$Pseudo\;R^{2}/R^{2}$	.102		.083		.074	
N	17,548		17,548		17,548	

*Note.* All models include sex and the following control variables: age group, visible minority status, marital status, presence of children, education, employment status, household income, and region.

All estimates are weighted. ***p < .001; **p < .01; *p < .05.

**Table 3.** Logistic and OLS regression models predicting self-rated health, mental health, and life satisfaction.

	Self-rated health				Self-rated mental health				Self-rated life satisfaction			
	Model 1		Model 2		Model 3		Model 4		Model 5		Model 6	
	OR	SE	OR	SE	OR	SE	OR	SE	B	SE	B	SE
Cyberbullying victimization (CV) (=1)	1.855**	.384	1.688	.635	1.415	.353	1.363	.511	.396***	.108	.541***	.149
Religious service attendance												
Not at all (reference)												
Once or twice a year	.875	.099			.912	.143			−.096*	.039		
At least three times a year	.885	.114			.736	.146			−.092	.048		
At least once a month	.977	.137			.628	.151			−.175***	.048		
At least once a week	.947	.098			.900	.150			−.192***	.044		
CV X religious service attendance												
CV X once or twice a year	1.692	.749			2.498	1.267			.344	.201		
CV X at least three times a year	1.358	.675			1.669	1.050			.222	.226		
CV X at least once a month	.968	.525			1.550	.849			−.017	.219		
CV X at least once a week	.244**	.113			.235	.198			−.659***	.200		
Importance of religious beliefs												
Not at all important (reference)												
Not very important			.828	.107			.810	.149			.060	.045
Somewhat important			.981	.106			.923	.144			−.025	.039
Very important			1.114	.119			1.355	.222			−.108*	.043
CV X importance of religious beliefs												
CV X not very important			1.412	.811			2.441	2.474			−.028	.233
CV X somewhat important			1.436	.673			1.666	.900			−.031	.191
CV X very important			.808	.360			.858	.402			−.398*	.202
Intercept	.180		.174		.047		.041		1.787		1.769	
$Pseudo\;R^{2}/R^{2}$	.104		.104		.086		.086		.079		.077	
N	17,548		17,548		17,548		17,548		17,548		17,548	

*Note.* All models include sex and the following control variables: age group, visible minority status, marital status, presence of children, education, employment status, household income, and region. All estimates are weighted. ***p < .001; **p < .01; *p < .05.

**Table 4.** Logistic and OLS regression models predicting self-rated health, mental health, and life satisfaction.

	Self-rated health				Self-rated mental health				Self-rated life satisfaction			
	Model 1		Model 2		Model 3		Model 4		Model 5		Model 6	
	OR	SE	OR	SE	OR	SE	OR	SE	B	SE	B	SE
Cyberbullying victimization (CV) (=1)	1.516	.494	.697	.332	1.190	.450	.849	.457	.330*	.147	.269	.189
Religious service attendance												
Not at all (reference)												
Once or twice a year	.965	.160			.710	.177			−.100	.055		
At least three times a year	1.194	.223			.691	.209			−.033	.072		
At least once a month	1.203	.254			.784	.318			−.125	.070		
At least once a week	1.247	.201			1.039	.283			−.241***	.069		
CV X religious service attendance												
CV X once or twice a year	1.164	.907			3.311	2.601			.102	.243		
CV X at least three times a year	2.376	1.632			3.770	3.485			.257	.358		
CV X at least once a month	1.701	1.291			1.784	1.382			−.066	.329		
CV X at least once a week	.212*	.168			.325	.373			−.625*	.314		
Women (=1)	1.210	.123	1.259	.208	1.062	.162	1.176	.287	−.041	.041	−.103	.060
CV X women	1.388	.591	4.111*	2.805	1.321	.656	2.138	1.562	.124	.213	.576*	.281
Religious service attendance X women												
Once or twice a year X women	.836	.190			1.520	.482			.007	.078		
At least three times a year X women	.570*	.146			1.111	.438			−.110	.096		
At least once a month X women	.687	.191			.679	.327			−.091	.095		
At least once a week X women	.619*	.126			.797	.266			.087	.086		
CV X religious service attendance X women												
CV X once or twice a year X women	1.822	1.742			.632	.654			.476	.384		
CV X at least three times a year X women	.274	.250			.205	.240			−.067	.457		
CV X at least once a month X women	.329	.334			.778	.853			.103	.432		
CV X at least once a week X women	1.341	1.284			.411	.604			−.037	.389		
Importance of religious beliefs												
Not at all important (reference)												
Not very important			.973	.170			.951	.244			.021	.059
Somewhat important			1.144	.173			.882	.198			−.028	.053
Very important			1.268	.193			1.539	.358			−.169**	.061
CV X importance of religious beliefs												
CV X not very important			4.439	3.393			4.203	3.523			.517	.309
CV X somewhat important			2.308	1.562			2.339	1.975			.002	.241
CV X very important			2.749	1.682			1.913	1.316			−.176	.287
Importance of religious beliefs X women												
Not very important X women			.699	.182			.727	.266			.094	.091
Somewhat important X women			.716	.152			1.048	.330			.025	.076
Very important X women			.755	.154			.792	.245			.125	.081
CV X importance of religious beliefs X women												
CV X not very important X women			.127*	.132			.401	.486			−1.114*	.449
CV X somewhat important X women			.489	.440			.578	.625			−.117	.364
CV X very important X women			.125*	.103			.249	.232			−.479	.393
Intercept	.162		.158		.047		.039		1.790		1.800	
$Pseudo\;R^{2}/R^{2}$	.107		.106		.088		.088		.080		.078	
N	17,548		17,548		17,548		17,548		17,548		17,548	

*Note.* All models include sex and the following control variables: age group, visible minority status, marital status, presence of children, education, employment status, household income, and region. All estimates are weighted. ***p<.001; **p<.01; *p<.05.

## Results

### Cyberbullying Victimization and Self-rated Health, Mental Health, and Life Satisfaction

Table 2 presents logistic and OLS regression models predicting self-rated health, mental health, and life satisfaction. Model 1 tests the direct association between cyberbullying victimization and self-rated health, finding that adults who experienced cyberbullying victimization in the past 5 years are more likely to report poor self-rated health compared to those who did not experience cyberbullying victimization in the past 5 years (OR = 1.872, SE = .294, *p* < .001). Similar patterns have also been observed for self-rated mental health and life satisfaction, suggesting that adults who experienced cyberbullying victimization in the past 5 years are more likely to report poor self-rated mental health (OR = 1.761, SE = .348, *p* < .01) and are associated with poorer self-rated life satisfaction (b = .395, SE = .074, *p* < .001) compared to those who did not experience cyberbullying victimization in the past 5 years. Together, these findings support Hypothesis 1.

### Moderating Effect of Religiosity

Table 3 presents logistic and OLS regression models predicting the moderating role of religiosity in the association between cyberbullying victimization and health and well-being outcomes. Model 1 tests the moderating effect of religious service attendance, suggesting that the adverse association of cyberbullying victimization in the past 5 years with self-rated health is weaker among adults who attended religious service at least once a week in the past twelve months compared to their counterparts who did not attend religious service in the past twelve months (OR = .244, SE = .113, *p* < .01 for cyberbullying victimization X at least once a week). However, as shown in Model 2, there is little evidence that the importance of religious beliefs moderates the association between cyberbullying victimization and self-rated health.

Model 3 tests the moderating effect of religious service attendance in the association between cyberbullying victimization and self-rated mental health. The interaction is not statistically significant. Likewise, Model 4 tests the moderating effect of the importance of religious beliefs, and the interaction is also not statistically significant. These results suggest that neither religious service attendance nor the importance of religious beliefs moderate the association between cyberbullying victimization and self-rated mental health.

Model 5 tests the moderating effect of religious service attendance in the association between cyberbullying victimization and self-rated life satisfaction, showing that the adverse association of cyberbullying victimization in the past 5 years with self-rated life satisfaction is weaker among adults who attended religious service at least once a week in the past twelve months compared to their counterparts who did not attend religious service in the past twelve months (b = −.659, SE = .200, *p* < .001 for cyberbullying victimization X at least once a week). Model 6 tests the moderating effect of the importance of religious beliefs, finding that the adverse association of cyberbullying victimization in the past 5 years with self-rated life satisfaction is weaker among adults who view religious beliefs as very important compared to their counterparts who do not view religious beliefs as important at all (b = −.398, SE = .202, *p* < .05 for cyberbullying victimization X very important). Together, these results partially support Hypothesis 2.

### Gender Differences

Table 4 presents logistic and OLS regression models predicting gender differences of the moderating effect of religiosity in the association between cyberbullying victimization and health and well-being outcomes. Model 1 tests whether the buffering effect of religious service attendance in the association between cyberbullying victimization and self-rated health observed in Table 3 further differs for men and women. The three-way interaction is not statistically significant, which does not suggest the gendered moderating effect of religious service attendance.

Although the importance of religious beliefs does not modify the effect of cyberbullying victimization on self-rated health, as shown in Table 3, it is still possible that its buffering effect might differ for men and women. Model 2 tests the three-way interaction between cyberbullying victimization, the importance of religious beliefs, and gender. The interaction is statistically significant (OR = .127, SE = .132, *p* < .05 for cyberbullying victimization X not very important X women; OR = .125, SE = .103, *p* < .05 for cyberbullying victimization X very important X women). In ancillary analyses (Appendix 1), I tested the interaction between cyberbullying victimization and the importance of religious beliefs for men and women, separately, finding that while the interaction between cyberbullying victimization and not very important is statistically significant for men (OR = 5.231, SE = 4.009, *p* < .05), it is not statistically significant for women (OR = .541, SE = .371, *p* > .05). However, given that the standard error of the interaction is extremely large for men (i.e., SE = 4.009), the estimate (i.e., OR = 5.231) is less likely to be meaningful.

Appendix 1 also reveals that, while the interaction between cyberbullying victimization and very important is not statistically significant for men (OR = 2.712, SE = 1.669, *p* > .05), it is statistically significant for women (OR = .347, SE = .190, *p* < .10). These findings suggest that the importance of religious beliefs moderates the adverse association between cyberbullying victimization and self-rated health for women (i.e., the detrimental effect of cyberbullying victimization in the past 5 years on self-rated health is weaker among women who view religious beliefs as very important compared to their counterparts who do not view religious beliefs as important at all), but not for men, and that differences are statistically significant, as suggested by the three-way interaction (Model 2 in Table 4).

Likewise, although neither religious service attendance nor the importance of religious beliefs modify the effect of cyberbullying victimization on self-rated mental health, as shown in Table 3, it is still possible that their buffering effects might differ for men and women. Models 3 and 4 test three-way interactions between cyberbullying victimization, religiosity, and gender. Model 3 shows that the three-way interaction is not statistically significant, which does not suggest the gendered moderating effect of religious service attendance. Similarly, Model 4 shows that the three-way interaction is not statistically significant, which does not suggest the gendered moderating effect of the importance of religious beliefs.

Model 5 tests whether the buffering effect of religious service attendance in the adverse association between cyberbullying victimization and self-rated life satisfaction observed in Table 3 further differs for men and women. The three-way interaction is not statistically significant, which does not suggest the gendered moderating effect of religious service attendance.

Model 6 tests whether the buffering effect of the importance of religious beliefs in the adverse association between cyberbullying victimization and self-rated life satisfaction observed in Table 3 further differs for men and women. The three-way interaction is statistically significant (B = −1.114, SE = .449, *p* < .05 for cyberbullying victimization X not very important X women). In ancillary analyses (Appendix 2), I tested the interaction between cyberbullying victimization and the importance of religious beliefs for men and women, separately, finding that this interaction was marginally significant for men (B = .530, SE = .309, *p* < .10) and for women (B = −.597, SE = .324, *p* < .10). These differences between men and women are large enough to be statistically significant, as suggested by the three-way interaction (Model 6 in Table 4), suggesting that the importance of religious beliefs buffers the adverse association of cyberbullying victimization with self-rated life satisfaction for women (i.e., the detrimental effect of cyberbullying victimization in the past 5 years is weaker on self-rated life satisfaction among women who view religious beliefs as not very important compared to their counterparts who do not view religious beliefs as important at all), but not for men.

Moreover, as shown in Appendix 2, while the interaction between cyberbullying victimization and very important is not statistically significant for men (B = −.167, SE = .288, *p* > .05), it is statistically significant for women (B = −.629, SE = .269, *p* < .05). However, these differences between men and women are not larger enough to be statistically significant, as suggested by the three-way interaction (Model 6 in Table 4). Taken together, these findings based on Table 4 partially support Hypothesis 3.

## Discussion

Cyberbullying has emerged as a significant public health concern affecting children and adolescents (Chester et al., 2019). Although prior research has well established the detrimental effect of cyberbullying victimization on health and well-being among children and adolescents (Müller et al., 2018; Parris et al., 2020; Quintana-Orts et al., 2020; Zhang et al., 2020; Zhu et al., 2019), less is known about the extent to which cyberbullying victimization shapes adults’ health and well-being. Moreover, although a growing body of work links religion to health (Das & Nairn, 2016; Elliott & Hayward, 2009), there has been little research that interests in the interaction of cyberbullying victimization and religion in shaping multiple health and well-being consequences. Thus, the present study advances current understanding of cyberbullying by: (a) examining the effect of cyberbullying victimization on adults’ self-rated health, mental health, and life satisfaction using a nationally representative Canadian sample; and (b) exploring the moderating potential of two measures of religiosity—religious service attendance and the importance of religious beliefs—in the association between cyberbullying victimization and health and well-being.

Several findings are noteworthy. First, I found that adults who experienced cyberbullying victimization in the past 5 years were more likely to report poor self-rated health and mental health and were associated with lower levels of life satisfaction compared to those who did not experience cyberbullying victimization in the past 5 years. These patterns are consistent with prior research focusing on children and adolescents. Many past studies that document the consequences of cyberbullying victimization among adults have focused on work outcomes, such as job satisfaction, absenteeism, and intention to resign (Baruch, 2005; Coyne et al., 2017; Farley et al., 2015; Kowalski et al., 2018; Snyman & Loh, 2015), though there is preliminary evidence that cyberbullying victimization is positively linked to mental health and substance use problems (Kim et al., 2017). The present study provides additional empirical evidence on self-rated health and life satisfaction, confirming that the adverse association between cyberbullying victimization and health and well-being does not restrict to children and adolescents.

Second, as one of the first studies to assess the moderating potential of religion in the context of cyberbullying victimization, I found evidence supporting this claim. For religious service attendance, the results showed that the adverse associations of cyberbullying victimization in the past 5 years with self-rated health and life satisfaction were weaker among those who attended religious service at least once a week in the past twelve months. These patterns confirm the proposition that attending a religious congregation might provide benefits for people who experienced cyberbullying victimization. For instance, given that religious congregations often promote health behaviors such as vitamin use, infrequent bar attendance, never smoking, and moderate drinking (Hill et al., 2006), victims of cyberbullying might improve healthy eating and monitor their drinking or/and smoking habits, thereby contributing to better health consequences. In addition, social and friendship support from the fellow community members might also help individuals who encountered cyberbullying victimization recover from stressful experiences.

In addition, the results also showed that the importance of religious beliefs lessened the deleterious effect of cyberbullying victimization on life satisfaction. That is, the adverse association of cyberbullying victimization in the past 5 years with life satisfaction was weaker among those who viewed religious beliefs as very important. This finding aligns with past research that developing a personal connection with a higher power allows individuals who experienced stressful events or chronic stressors to feel a sense of comfort and reassurance because knowing that all the adverse encounters might be planned by God (Bradshaw & Ellison, 2010; Jung, 2014), thereby weakening the adverse effect of social stressors.

Nevertheless, there is little evidence suggesting the same buffering patterns of religious service attendance for self-rated mental health and patterns of the importance of religious beliefs for self-rated mental health and health. The statistically insignificant results for self-rated mental health might be attributed to the large standard error due to the small cell size. Potentially as a result of social desirability biases, only 4% (i.e., 761 adults) of respondents reported “fair/poor” mental health, which are further divided when testing the moderating effect of religiosity (i.e., religious service attendance and the importance of religious beliefs) in the association between cyberbullying victimization and self-rated mental health, which might have decreased the power to detect significant associations. Future research should re-assess these moderating associations using a continuous measure of mental health. Moreover, the statistically insignificant result for self-rated health might suggest that religious service attendance is more practical than religious beliefs in shaping cyberbullying victims’ overall health outcome. Nevertheless, more investigations are warranted given the scarce research on the moderating potential of the importance of religious beliefs.

Third, based on the three-way interaction, I found that the buffering effect of the importance of religious beliefs was gendered in the association between cyberbullying victimization and poor self-rated health. That is, viewing religious beliefs as very important marginally buffered the detrimental effect of cyberbullying for women, but not for men. Together, these findings are consistent with the prediction of gender role socialization perspective, suggesting that women benefit more from religion compared to men (Jung, 2014).

Despite its contributions, the study also has several limitations. First, the Canadian General Social Survey data is cross-sectional, which limits the capacity to document the potential causal direction of these relationships. In addition to replicating cross-sectional findings presented in this study, it would be important for future studies to explore the patterns observed here using longitudinal data. Second, the GSS does not measure other forms of bullying behaviors (e.g. traditional bullying), which prevents me from confirming whether the findings observed in the present study apply to other forms of bullying behaviors as well. Third, the GSS does not have variables related to peer, family, and community, which might affect cyberbullying victimization. As Kim and colleagues (2017) stressed, “future studies that encompass a broader social-ecological perspective of [cyberbullying victimization] would be beneficial to help inform the development of comprehensive preventive intervention programs” (p. e473).

## Conclusion

Despite these limitations, this study makes important contributions to the literature on cyberbullying victimization, religion, and health among adults. First, the study demonstrates that adults are not immune to cyberbullying victimization by showing its detrimental effect of on self-rated health, mental health, and life satisfaction. Therefore, future studies on health disparities by cyberbullying victimization need to go beyond the scope of analyzing children and adolescents. Second, the study reveals that the buffering effect of religion depends on the specific health and well-being outcome being examined. To provide a broader picture, it is important for future research to document multiple health and well-being outcomes simultaneously. Together, the study is among the first that formally assesses the moderating potential of religion in the association between cyberbullying victimization and health and well-being using a nationally representative sample. Future research on cyberbullying victimization and adults’ health and well-being should explore the extent to which other moderating potentials that might weaken the detrimental effect of cyberbullying victimization.

## Appendix 1

### Logistic regression models predicting self-rated health for men and women.

**Table:**

	Self-rated health			
	Model 1: men		Model 2: women	
	OR	SE	OR	SE
Cyberbullying victimization (CV) (=1)	.731	.349	2.632*	1.271
Importance of religious beliefs				
Not at all important (reference)				
Not very important	.941	.165	.690+	.133
Somewhat important	1.091	.164	.858	.133
Very important	1.202	.185	1.026	.151
CV X importance of religious beliefs				
CV X not very important	5.231*	4.009	.541	.371
CV X somewhat important	2.468	1.666	1.102	.663
CV X very important	2.712	1.669	.347+	.190
Intercept	.158		.188	
$Pseudo\;R^{2}$	.107		.112	
N	7769		9779	

*Note.* All models include the following control variables: age group, visible minority status, marital status, presence of children, education, employment status, household income, and region. All estimates are weighted. ****p*<.001; ***p*<.01; **p*<.05; +*p*<.10.

## Appendix 2

### OLS regression models predicting self-rated life satisfaction for men and women.

**Table:**

	Self-rated life satisfaction			
	Model 1: men		Model 2: women	
	B	SE	B	SE
Cyberbullying victimization (CV) (=1)	.297	.189	.808***	.208
Importance of religious beliefs				
Not at all important (reference)				
Not very important	.010	.059	.115+	.069
Somewhat important	−.041	.053	.011	.056
Very important	−.199**	.063	−.016	.060
CV X importance of religious beliefs				
CV X not very important	.530+	.309	−.597+	.324
CV X somewhat important	−.029	.241	−.111	.270
CV X very important	−.167	.288	−.629*	.269
Intercept	1.900		1.597	
$R^{2}$	.079		.084	
N	7769		9779	

*Note.* All models include the following control variables: age group, visible minority status, marital status, presence of children, education, employment status, household income, and region. All estimates are weighted. ****p*<.001; ***p*<.01; **p*<.05; +*p*<.10.

## References

[bibr1-08862605211050092] Aboujaoude E. Savage M. W. Starcevic V. Salame W. O. (2015). Cyberbullying: Review of an old problem gone viral. Journal of Adolescent Health, 57(1), 10–18. 10.1016/j.jadohealth.2015.04.011 26095405

[bibr2-08862605211050092] Ahmad F. Jhajj A. K. Stewart D. E. Burghardt M. Bierman A. S. (2014). Single item measures of self-rated mental health: A scoping review. BMC Health Services Research, 14(1), 398–411. 10.1186/1472-6963-14-398 25231576 PMC4177165

[bibr3-08862605211050092] Baruch Y. (2005). Bullying on the net: adverse behavior on e-mail and its impact. Information & Management, 42(2), 361–371. 10.1016/j.im.2004.02.001

[bibr4-08862605211050092] Bennett K. M. (2007). “No sissy stuff”: Towards a theory of masculinity and emotional expression in older widowed men. Journal of Aging Studies, 21(4), 347–356. 10.1016/j.jaging.2007.05.002

[bibr5-08862605211050092] Bierman A. (2006). Does religion buffer the effects of discrimination on mental health? Differing effects by race. Journal for the Scientific Study of Religion, 45(4), 551–565. 10.1111/j.1468-5906.2006.00327.x

[bibr6-08862605211050092] Bierman A. Lee Y. Schieman S. (2018). Chronic discrimination and sleep problems in late life: Religious involvement as buffer. Research on Aging, 40(10), 933–955. 10.1177/0164027518766422 29580186

[bibr7-08862605211050092] Bonanno R. A. Hymel S. (2013). Cyber bullying and internalizing difficulties: Above and beyond the impact of traditional forms of bullying. Journal of Youth and Adolescence, 42(5), 685–697. 10.1007/s10964-013-9937-1. 23512485

[bibr8-08862605211050092] Bradshaw M. Ellison C. G. (2010). Financial hardship and psychological distress: Exploring the buffering effects of religion. Social Science & Medicine, 71(1), 196–204. 10.1016/j.socscimed.2010.03.015 20556889 PMC3770858

[bibr9-08862605211050092] Chai L. Xue J. (2021). Weight, weight perceptions, and health and well-being among Canadian adolescents: Evidence from the 2017-2018 Canadian community health survey. American Journal of Health Promotion. 10.1177/08901171211031064 PMC8669201 34282629

[bibr10-08862605211050092] Chai L. Xue J. Han Z. (2020). School bullying victimization and self-rated health and life satisfaction: The gendered buffering effect of educational expectations. Children and Youth Services Review, 116(C), 105252. 10.1016/j.childyouth.2020.105252

[bibr11-08862605211050092] Chester K. L. Magnusson J. Klemera E. Spencer N. H. Brooks F. (2019). The mitigating role of ecological health assets in adolescent cyberbullying victimization. Youth & Society, 51(3), 291–317. 10.1177/0044118X16673281

[bibr12-08862605211050092] Chu X.-W. Fan C.-Y. Liu Q.-Q. Zhou Z.-K. (2018). Cyberbullying victimization and symptoms of depression and anxiety among Chinese adolescents: Examining hopelessness as a mediator and self-compassion as a moderator. Computers in Human Behavior, 86, 377-386. 10.1016/j.chb.2018.04.039.

[bibr13-08862605211050092] Cole D. A. Zelkowitz R. L. Nick E. Martin N. C. Roeder K. M. Sinclair-McBride K. Spinelli T. (2016). Longitudinal and incremental relation of cybervictimization to negative self-cognitions and depressive symptoms in young adolescents. Journal of Abnormal Child Psychology, 44(7), 1321–1332. 10.1007/s10802-015-0123-7 26747449 PMC4938781

[bibr14-08862605211050092] Coyne I. Farley S. Axtell C. Sprigg C. Best L. Kwok O. (2017). Understanding the relationship between experiencing workplace cyberbullying, employee mental strain and job satisfaction: A dysempowerment approach. The International Journal of Human Resource Management, 28(7), 945–972. 10.1080/09585192.2015.1116454

[bibr15-08862605211050092] Das A. Nairn S. (2016). Religious attendance and physiological problems in late life. The Journals of Gerontology Series B: Psychological Sciences and Social Sciences, 71(2), 291–308. 10.1093/geronb/gbu089 25098525

[bibr16-08862605211050092] DeSalvo K. B. Bloser N. Reynolds K. He J. Muntner P. (2006). Mortality prediction with a single general self-rated health question. Journal of General Internal Medicine, 21(3), 267–275. 10.1111/j.1525-1497.2005.00291.x 16336622 PMC1828094

[bibr17-08862605211050092] Elliott M. Hayward R. D. (2009). Religion and life satisfaction worldwide: The role of government regulation. Sociology of Religion, 70(3), 285–310. 10.1093/socrel/srp028

[bibr18-08862605211050092] Ellison C. G. Deangelis R. T. Hill T. D. Froese P. (2019). Sleep quality and the stress-buffering role of religious involvement: A mediated moderation analysis. Journal for the Scientific Study of Religion, 58(1), 251–268. 10.1111/jssr.12581

[bibr19-08862605211050092] Farley S. Coyne I. Sprigg C. Axtell C. Subramanian G. (2015). Exploring the impact of workplace cyberbullying on trainee doctors. Medical Education, 49(4), 436–443. 10.1111/medu.12666 25800304

[bibr20-08862605211050092] Ferraro K. F. Farmer M. M. Wybraniec J. A. (1997). Health trajectories: Long-term dynamics among black and white adults. Journal of Health and Social Behavior, 38(1), 38–54. 10.2307/2955360 9097507

[bibr21-08862605211050092] Fisher B. W. Gardella J. H. Teurbe-Tolon A. R. (2016). Peer cybervictimization among adolescents and the associated internalizing and externalizing problems: A meta-analysis. Journal of Youth and Adolescence, 45(9), 1727–1743. 10.1007/s10964-016-0541-z 27447707

[bibr22-08862605211050092] Gorsuch R. L. Hao J. Y. (1993). Forgiveness: An exploratory factor analysis and its relationships to religious variables. Review of Religious Research, 34(4), 333-347. 10.2307/3511971.

[bibr23-08862605211050092] Hastings O. P. Roeser K. K. (2020). Happiness in hard times: Does religion buffer the negative effect of unemployment on happiness? Social Forces, 99(2), 447–473. 10.1093/sf/soaa018

[bibr24-08862605211050092] Hill T. D. Burdette A. M. Ellison C. G. Musick M. A. (2006). Religious attendance and the health behaviors of Texas adults. Preventive Medicine, 42(4), 309–312. 10.1016/j.ypmed.2005.12.005 16445971

[bibr25-08862605211050092] Hinduja S. Patchin J. W. (2019). Connecting adolescent suicide to the severity of bullying and cyberbullying. Journal of School Violence, 18(3), 333–346. 10.1080/15388220.2018.1492417

[bibr26-08862605211050092] Huang Y.-C. Zuñiga J. García A. García A. (2021). Emotional distress and self-rated health among middle-aged and older Chinese Americans with Type 2 diabetes. Journal of Immigrant and Minority Health, 23(3), 487–493. 10.1007/s10903-020-01062-x. 32748103

[bibr27-08862605211050092] Idler E. L. Benyamini Y. (1997). Self-rated health and mortality: A review of twenty-seven community studies. Journal of Health and Social Behavior, 38(1), 21–37. 10.2307/2955359 9097506

[bibr28-08862605211050092] Jenaro C. Flores N. Frías C. P. (2021). Anxiety and depression in cyberbullied college students: A retrospective study. Journal of Interpersonal Violence, 36(1-2), 579–602. 10.1177/0886260517730030 29294905

[bibr29-08862605211050092] Jovanović V. Lazić M. (2020). Is longer always better? A comparison of the validity of single-item versus multiple-item measures of life satisfaction. Applied Research in Quality of Life, 15, 675-692. 10.1007/s11482-018-9680-6.

[bibr30-08862605211050092] Jung J. H. (2014). Religious attendance, stress, and happiness in South Korea: Do gender and religious affiliation matter? Social Indicators Research, 118(3), 1125–1145. 10.1007/s11205-013-0459-8

[bibr67-08862605211050092] Jung J. H. (2020). Does religious bonding moderate the association between divine struggles and depressive symptoms among married adults? Psychology of Religion and Spirituality, 12(3), 376-386. 10.1037/rel0000308.

[bibr31-08862605211050092] Jung J. H. (2021). Perceived distributive unfairness and mental health: The gender-contingent buffering effects of religion. Society and Mental Health. 10.1177/2156869320978793

[bibr32-08862605211050092] Jylhä M. (2009). What is self-rated health and why does it predict mortality? Towards a unified conceptual model. Social Science & Medicine, 69(3), 307–316. 10.1016/j.socscimed.2009.05.013 19520474

[bibr33-08862605211050092] Kim S. Boyle M. H. Georgiades K. (2017). Cyberbullying victimization and its association with health across the life course: A Canadian population study. Canadian Journal of Public Health, 108(5-6), e468–e474. 10.17269/CJPH.108.6175 PMC6972207 29356651

[bibr34-08862605211050092] Kowalski R. M. Giumetti G. W. Schroeder A. N. Lattanner M. R. (2014). Bullying in the digital age: A critical review and meta-analysis of cyberbullying research among youth. Psychological Bulletin, 140(4), 1073–1137. 10.1037/a0036634 24512111

[bibr35-08862605211050092] Kowalski R. M. Limber S. P. (2013). Psychological, physical, and academic correlates of cyberbullying and traditional bullying. Journal of Adolescent Health, 53(1), S13–S20. 10.1016/j.jadohealth.2012.09.018 23790195

[bibr36-08862605211050092] Kowalski R. M. Toth A. Morgan M. (2018). Bullying and cyberbullying in adulthood and the workplace. The Journal of Social Psychology, 158(1), 64–81. 10.1080/00224545.2017.1302402 28402201

[bibr37-08862605211050092] Krause N. (2005). God-mediated control and psychological well-being in late life. Research on Aging, 27(2), 136-164. 10.1177/0164027504270475.

[bibr38-08862605211050092] Krause N. Ellison C. G. Marcum J. P. (2002). The effects of church-based emotional support on health: Do they vary by gender? Sociology of Religion, 63(1), 21–47. 10.2307/3712538

[bibr39-08862605211050092] Martínez-Monteagudo M. C. Delgado B. Díaz-Herrero Á. García-Fernández J. M. (2020). Relationship between suicidal thinking, anxiety, depression and stress in university students who are victims of cyberbullying. Psychiatry Research, 286, 112856. 10.1016/j.psychres.2020.112856 32062285

[bibr40-08862605211050092] McFarland M. J. (2010). Religion and mental health among older adults: Do the effects of religious involvement vary by gender? The Journals of Gerontology Series B: Psychological Sciences and Social Sciences, 65B(5), 621–630. 10.1093/geronb/gbp112 PMC2920941 20007301

[bibr41-08862605211050092] Mitchell S. M. Seegan P. L. Roush J. F. Brown S. L. Sustaíta M. A. Cukrowicz K. C. (2018). Retrospective cyberbullying and suicide ideation: The mediating roles of depressive symptoms, perceived burdensomeness, and thwarted belongingness. Journal of Interpersonal Violence, 33(16), 2602–2620. 10.1177/0886260516628291 26862162

[bibr42-08862605211050092] Müller C. R. Pfetsch J. Schultze-Krumbholz A. Ittel A. (2018). Does media use lead to cyberbullying or vice versa? Testing longitudinal associations using a latent cross-lagged panel design. Computers in Human Behavior, 81, 93–101. 10.1016/j.chb.2017.12.007

[bibr43-08862605211050092] Parris L. Lannin D. G. Hynes K. Yazedjian A. (2020). Exploring social media rumination: Associations with bullying, cyberbullying, and distress. Journal of Interpersonal Violence. 10.1177/0886260520946826 32757811

[bibr44-08862605211050092] Patchin J. W. Hinduja S. (2011). Traditional and nontraditional bullying among youth: A test of general strain theory. Youth & Society, 43(2), 727–751. 10.1177/0044118X10366951

[bibr45-08862605211050092] Payne A. A. Hutzell K. L. (2017). Old wine, new bottle? Comparing interpersonal bullying and cyberbullying victimization. Youth & Society, 49(8), 1149–1178. 10.1177/0044118X15617401

[bibr46-08862605211050092] Pearlin L. I. Bierman A. (2013). Current issues and future directions in research into the stress process. In Aneshensel C. Phelan J. Bierman A. (Eds), Handbook of the sociology of mental health (pp. 325–340). Springer

[bibr47-08862605211050092] Perret L. C. Orri M. Boivin M. Ouellet‐Morin I. Denault A. S. Côté S. M. Renaud J. Turecki G. Geoffroy M. C. (2020). Cybervictimization in adolescence and its association with subsequent suicidal ideation/attempt beyond face‐to‐face victimization: A longitudinal population‐based study. Journal of Child Psychology and Psychiatry, 61(8), 866–874. 10.1111/jcpp.13158 32017089

[bibr48-08862605211050092] Quintana-Orts C. Rey L. Neto F. (2020). Beyond cyberbullying: Investigating when and how cybervictimization predicts suicidal ideation. Journal of Interpersonal Violence. 10.1177/0886260520913640 32345110

[bibr49-08862605211050092] Ranney M. L. Patena J. V. Nugent N. Spirito A. Boyer E. Zatzick D. Cunningham R. (2016). PTSD, cyberbullying and peer violence: Prevalence and correlates among adolescent emergency department patients. General Hospital Psychiatry, 39, 32–38. 10.1016/j.genhosppsych.2015.12.002 26786845 PMC4779373

[bibr50-08862605211050092] Salazar R. L. (2017). Cyberbullying victimization as a predictor of cyberbullying perpetration, body image dissatisfaction, healthy eating and dieting behaviors, and life satisfaction. Journal of Interpersonal Violence, 36(4), 088626051772573. 10.1177/0886260517725737 29294894

[bibr51-08862605211050092] Schneider S. K. O’donnell L. Stueve A. Coulter R. W. S. (2012). Cyberbullying, school bullying, and psychological distress: A regional census of high school students. American Journal of Public Health, 102(1), 171–177. 10.2105/AJPH.2011.300308 22095343 PMC3490574

[bibr52-08862605211050092] Shah S. (2019). Does religion buffer the effects of discrimination on distress for religious Minorities? The case of Arab Americans. Society and Mental Health, 9(2), 171–191. 10.1177/2156869318799145

[bibr53-08862605211050092] Sivakumaran G. Margolis R. (2020). Self-rated health by sexual orientation among middle-aged and older adults in Canada. The Journals of Gerontology: Series B, 75(8), 1747–1757. 10.1093/geronb/gbz067 PMC7489081 31120125

[bibr54-08862605211050092] Snyman R. Loh J. (2015). Cyberbullying at work: The mediating role of optimism between cyberbullying and job outcomes. Computers in Human Behavior, 53, 161–168. 10.1016/j.chb.2015.06.050

[bibr55-08862605211050092] Statistics Canada (2014). General social survey: An overview, 2014: Minister of Industry.

[bibr56-08862605211050092] Tokunaga R. S. (2010). Following you home from school: A critical review and synthesis of research on cyberbullying victimization. Computers in Human Behavior, 26(3), 277–287. 10.1016/j.chb.2009.11.014

[bibr57-08862605211050092] Torres C. E. D’Alessio S. J. Stolzenberg L. (2020). The effect of social, verbal, physical, and cyberbullying victimization on academic performance. Victims & Offenders, 15(1), 1–21. 10.1080/15564886.2019.1681571

[bibr58-08862605211050092] Vaillancourt T. Faris R. Mishna F. (2017). Cyberbullying in children and youth: Implications for health and clinical practice. The Canadian Journal of Psychiatry, 62(6), 368–373. 10.1177/0706743716684791 28562091 PMC5455867

[bibr59-08862605211050092] Vieno A. Gini G. Lenzi M. Pozzoli T. Canale N. Santinello M. (2015). Cybervictimization and somatic and psychological symptoms among Italian middle school students. The European Journal of Public Health, 25(3), 433–437. 10.1093/eurpub/cku191 25465914

[bibr60-08862605211050092] Waasdorp T. E. Bradshaw C. P. (2015). The overlap between cyberbullying and traditional bullying. Journal of Adolescent Health, 56(5), 483–488. 10.1016/j.jadohealth.2014.12.002 25631040

[bibr61-08862605211050092] Wang L. (2021). The effects of cyberbullying victimization and personality characteristics on adolescent mental health: An application of the stress process model. Youth & Society. 10.1177/0044118X211008927

[bibr62-08862605211050092] Wang C. W. Musumari P. M. Techasrivichien T. Suguimoto S. P. Tateyama Y. Chan C. C. Ono-Kihara M. Kihara M. Nakayama T. (2019). Overlap of traditional bullying and cyberbullying and correlates of bullying among Taiwanese adolescents: A cross-sectional study. BMC Public Health, 19(1), 1756–1814. 10.1186/s12889-019-8116-z 31888598 PMC6937625

[bibr63-08862605211050092] Wang W. Xie X. Wang X. Lei L. Hu Q. Jiang S. (2019). Cyberbullying and depression among Chinese college students: A moderated mediation model of social anxiety and neuroticism. Journal of Affective Disorders, 256, 54–61. 10.1016/j.jad.2019.05.061 31158716

[bibr64-08862605211050092] Wu Z. Schimmele C. M. (2021). Perceived religious discrimination and mental health. Ethnicity & Health, 26(7), 963-980. 10.1080/13557858.2019.1620176. 31117819

[bibr65-08862605211050092] Zhang D. Huebner E. S. Tian L. (2020). Longitudinal associations among neuroticism, depression, and cyberbullying in early adolescents. Computers in Human Behavior, 112, 106475. 10.1016/j.chb.2020.106475

[bibr66-08862605211050092] Zhu Y. Li W. O’Brien J. E. Liu T. (2019). Parent–child attachment moderates the associations between cyberbullying victimization and adolescents’ health/mental health problems: An exploration of cyberbullying victimization among Chinese adolescents. Journal of Interpersonal Violence. 10.1177/0886260519854559 31200608

